# An Update on Resistance Genes and Their Use in the Development of Leaf Rust Resistant Cultivars in Wheat

**DOI:** 10.3389/fgene.2022.816057

**Published:** 2022-03-31

**Authors:** Kuldeep Kumar, Irfat Jan, Gautam Saripalli, P. K. Sharma, Reyazul Rouf Mir, H. S. Balyan, P. K. Gupta

**Affiliations:** ^1^ Department of Genetics and Plant Breeding, Chaudhary Charan Singh University, Meerut, India; ^2^ Division of Genetics and Plant Breeding, Faculty of Agriculture, Sher-e-Kashmir University of Agricultural Sciences and Technology, Wadura, India; ^3^ Department of Plant Science and Landscape Architecture, University of Maryland, College Park, MD, United States

**Keywords:** bread wheat, leaf rust, genes, QTLs, markers, molecular breeding

## Abstract

Wheat is one of the most important cereal crops in the world. The production and productivity of wheat is adversely affected by several diseases including leaf rust, which can cause yield losses, sometimes approaching >50%. In the present mini-review, we provide updated information on (i) all Lr genes including those derived from alien sources and 14 other novel resistance genes; (ii) a list of QTLs identified using interval mapping and MTAs identified using GWAS (particular those reported recently i.e., after 2018) and their association with known Lr genes; (iii) introgression/pyramiding of individual Lr genes in commercial/prominent cultivars from 18 different countries including India. Challenges and future perspectives of breeding for leaf rust resistance are also provided at the end of this mini-review. We believe that the information in this review will prove useful for wheat geneticists/breeders, not only in the development of leaf rust-resistant wheat cultivars, but also in the study of molecular mechanism of leaf rust resistance in wheat.

## Introduction

Leaf rust caused by the fungal pathogen *Puccinia triticina* Eriks. & E. Henn is an important disease in wheat, which causes significant yield losses, sometimes approaching up to >50% (Riaz and Wong 2017). The study of the genetic basis of this disease and breeding for leaf rust resistance in wheat has been an important area of research (Dyck 1993; Kolmer and Liu, 2002; Oelke and Kolmer 2005; Datta et al., 2008; Rosa et al., 2016; Aoun et al., 2017). Each individual Lr gene apparently shows resistance against a specific race of *P. triticina* (*Pt*), which must carry the corresponding avirulence (Avr) gene, such that a specific Lr gene in the host and the corresponding specific Avr gene in the pathogen always follow a “gene-for-gene” relationship (Flor 1946). The pathogen Pt keeps on developing new virulent races through mutations or recombination involving Avr genes; new strains may also migrate from other geographical areas, and may carry one or more new Avr genes for which the corresponding R gene may be absent in the host (Samborski 1985; Bolton et al., 2008). Therefore, the host resistance breaks down and is short-lived. It is thus obvious that a majority of race specific Lr genes individually do not provide durable resistance (Johnson 1984).

Lr genes provide either seedling resistance (SR), also described as all stage resistance (ASR), or adult plant resistance (APR genes), the latter expressed only at the adult plant stage, particularly after booting. It is also known that ASR genes provide resistance, which breaks down within a few years, while APR provides long-term durable resistance (Ellis et al., 2014). Some of the APR genes like *Lr34* and *Lr67* have also been cloned and were found to be complex loci including *Lr34*/*Sr57*/*Yr18*/*Pm38* and *Lr67*/*Sr55*/*Yr46*/*Pm46* (Lagudah et al., 2006; Moore et al., 2015). These gene complexes confer durable resistance not only against leaf rust, but also against stripe rust, stem rust, powdery mildew, and barley yellow dwarf virus (Singh and Rajaram, 1993). The use of APR genes along with 4–5 Lr genes is a strategy that provides durable resistance.

A number of reviews on leaf rust resistance in wheat have already been published (Kolmer 1996; Kolmer 2013; McCallum et al., 2016; Pinto da Silva et al., 2018; Dinh et al., 2020; Figlan et al., 2020; Ghimire et al., 2020; Prasad et al., 2020). Information about QTLs for leaf rust resistance has also been recently reviewed (Pinto da Silva et al., 2018). However, considerable literature has appeared during the last 3–4 years, where many more QTLs and as many as 600 new MTAs have been added thus warranting a fresh look on the subject, hence this minireview.

According to some recent reports, currently more than 80 Lr genes and 14 other genes for leaf rust resistance are known in wheat (McIntosh et al., 2017, 2020). The above 14 genes have not been assigned a new number in Lr series, perhaps because these genes have not been subjected to test of allelism with the known Lr genes to ascertain their novelty. Since literature on Lr genes keep on appearing on a regular basis, any review published soon becomes out of date thus creating the need for a fresh review. The present mini-review caters to this need and provides an updated information on all Lr genes and other genes including genes derived from alien species. The mini-review includes information about chromosomal location of all these genes (including 14 other resistance genes, which could not be assigned to any of the known Lr genes; modified names were used for these 14 genes based on the cultivar in which they were identified). We also provide information about the wild relatives of wheat as a source of Lr genes and the molecular markers associated with most of these genes (wherever known). Information about cloning and characterisation of Lr genes has also been included, wherever available. The wheat varieties carrying different Lr genes developed in 18 different countries are also listed.

## Lr Genes/Novel Lr Genes Catalogued so far

More than 80 Lr genes (∼50% derived from alien species) are already known to be distributed on all the 21 wheat chromosomes, with majority of genes located on the short arms of individual chromosomes (Table 1; Supplementary Tables S1, S2). Most of the Lr genes are located on the B sub-genome, relative to those located on either A sub-genome or D sub-genome. Maximum number of ten Lr genes (including two novel genes *LrZH22* and *LrG6*) are located on chromosome 2B. At least two of these genes, namely *Lr18* and *LrZH22*, are known to be temperature sensitive; *Lr18* exhibits resistance at 15–18°C, ineffective at >18°C (Carpenter et al., 2017). The other gene *LrZH22* confers resistance at higher temperatures (22–25°C; Wang et al., 2016). Lr genes conferring APR include the following: *Lr34, Lr46, Lr67, Lr68, Lr74, Lr75, Lr77* and *Lr78*. Information on QTLs/MTAs was also included in an earlier review (Pinto da Silva et al., 2018) and has been compiled by us also in this mini-review (Supplementary Tables S1, S2). A set of 14 novel resistance genes (including three genes from alien species) are known, which differ from other available Lr genes, since they show seedling reaction pattern, which was different from reaction patterns known for different Lr genes studied so far. These 14 genes along with associated markers are also listed in Table 1. These genes were mapped on 10 out of the 21 wheat chromosomes with maximum number of these genes available on B sub-genome (8) followed by sub-genome D (4) and sub-genome A (2).

**TABLE 1:** Details of leaf rust (Lr) resistant genes including novel Lr genes identified in bread wheat.

Gene	Chr	Marker		References
*Lr1*	5DL	psr567		Sylvie Cloutier et al. (2007)
*Lr2a*	2DS	Xwmc453 - XwPt0330		Tsilo et al. (2014)
*Lr3*	6BL	Xmwg798		Sacco et al. (1998)
*Lr3a*	6BL	UBC 840		Khan et al. (2005)
*Lr9*	6BL	SCS5		Gupta et al. (2005)
*Lr10*	1AS	Lrk10D1		Schachermayr et al. (1997)
*Lr11*	2DS	SCAR32/35		Darino et al. (2015)
*Lr12*	4BL	Xgwm251 - Xgwm149		Singh and Bowden (2011)
*Lr13*	2BS	Xbarc55-2B		Seyfarth et al. (1998); Seyfarth et al. (1999)
*Lr14a*	7BL	wPt-4038-HRM		Terracciano et al. (2013)
*Lr15*	2DS	Xgwm4562 - Xgwm102		Dholakia et al. (2013)
*Lr16*	2BS	Xwmc764, Xgwm210, and Xwmc661		McCartney et al. (2005)
*Lr17*	2AS	Xgwm614 - gwm407		Bremenkamp-Barrett et al. (2008)
*Lr18*	5BL	IWB41960 - gwm547		Carpenter et al. (2017)
*Lr19*	7DL	SCS265 and SCS253		Gupta et al. (2006)
*Lr20*	7AL	STS638		Neu et al. (2002)
*Lr21*	1DS	Lr21_GQ5044819_2175_G/A KASPar assay and Lr21_GQ5044819_3146_C/T KASPar assay		Neelam et al. (2013)
*Lr22a*	2DS	gwm296		Hiebert et al. (2007)
*Lr23*	2BS	Xtam72		Nelson et al. (1997)
*Lr24*	3DL	SCS1302		Prabhu et al. (2004)
*Lr25*	4BS	Xgwm251		Singh et al. (2012)
*Lr26*	1BL	P6M12-P		Mago et al. (2002), Zhou et al. (2014)
*Lr27*	3BS	cdo460		Nelson et al. (1997)
*Lr28*	4AL	SCS421		Naik et al. (1998), Cherukuri et al. (2005)
*Lr29*	7DS	ubc219		Procunier et al. (1995)
*Lr30*	4AL	IWA4359 - IWA2585		Aoun et al. (2019)
*Lr31*	4BL	XksuG10		Nelson et al. (1997)
*Lr32*	3DS	Xbcd1278		Autrique et al. (1995)
*Lr34*	7DS	csLV34		Lagudah et al. (2006, 2009)
*Lr35*	2BL	Xbcd260		Seyfarth et al. (1999)
*Lr36*	6BS	cfd1, gwm508		Dadkhodaie et al. (2011)
*Lr37*	2AS	VENTRIUP/LN2		Helguera et al. (2003)
*Lr38*	6DL	wmc773 - barc273		Mebrate et al. (2008)
*Lr39*	2DS	Xgwm210		Raupp et al. (2001)
*Lr41*	2DS	Xbarc124		Sun et al. (2009)
*Lr42*	1DS	Xwmc432		Sun et al. (2010)
*Lr45*	2AS	cfd168, G372 _94_ and G372 _185_		Naik et al. (2015)
*Lr46*	1BL	XSTS1BL9		Mateos-Hernandez et al. (2006)
*Lr47*	7AL	PS10		Helguera et al. (2000)
*Lr48*	2BL	Xksm58 - Xstm773-2		Bansal et al. (2008)
*Lr49*	4BL	Xbarc163 - Xwmc349		Bansal et al. (2008)
*Lr50*	2BL	Xgwm382		Brown-Guedira et al. (2003)
*Lr51*	1BL	e XAga7		Helguera et al. (2005)
*Lr52*	5BS	Xwmc149, Xtxw200		Tar et al. (2008)
*Lr53*	6BS	cfd1, gwm508		Dadkhodaie et al. (2011)
*Lr57*	5DS	Lr57/Yr40-MAS-CAPS16		Kuraparthy et al. (2009)
*Lr58*	2BL	Xcfd50		Kuraparthy et al. (2007)
*Lr59*	6BS	IWA1495, IWA6704		Poudel (2015)
*Lr60*	1DS	Xbarc149		Hiebert et al. (2008)
*Lr61*	6BS	P81/M70		Herrera-Foessel et al. (2008)
*Lr62*	6AS	Xgwm334		Marais et al. (2009)
*Lr63*	3AS	barc 57 and barc 321		Kolmer et al. (2010)
*Lr64*	6AS	K-IWB59855		Kolmer (2019)
*Lr65*	2AS	barc124, barc212, gwm614		Mohler et al. (2012)
*Lr66*	3AS	S13-R16		Marais et al. (2010)
*Lr67*	4DL	cfd71		Hiebert et al. (2010)
*Lr68*	7BL	Psy1-1 - gwm146		Herrera-Foessel et al. (2012)
*Lr70*	5DS	barc130		Hiebert et al. (2014)
*Lr71*	1BS	gwm18 - barc187		Singh et al. (2012)
*Lr72*	7BS	wmc606		Herrera-Foessel et al. (2014)
*Lr73*	2BL	wPt8760 - wPt-8235		Park et al. (2014)
*Lr74*	3BS	Xcfb5006 - Xgwm533		Li et al. (2017)
*Lr75*	1BS	gwm604 - swm271		Singla et al. (2017)
*Lr76*	5DL	*Lr57/Yr40*-MAS-CAPS16		Kuraparthy et al. (2009)
*Lr77*	3BL	IWB10344		Kolmer et al. (2018a)
*Lr78*	5DS	IWA6289		Kolmer et al. (2018b)
*Lr79*	3BL	sun786 - sun770		Qureshi et al. (2018)
*Lr80*	2DS	KASP_17425, KASP_17148		Kumar et al. (2021)
*LrX*	1DS	K-IWB38437		Kolmer et al. (2019)
*LrTs276-2*	1DS	Xcfd15 - Xcfd61		Dinkar et al. (2020)
*Lr2K38*	1AL	IWB20487		Sapkota et al. (2020)
**Novel Lr genes**				
*LrZH84*	1BL	Xgwm582 - Xbarc8		Zhao et al. (2008)
*LrBi16*	7BL	Xcfa2257		Zhang et al. (2011)
*LrSV1*	2DS	Xgwm261		Ingala et al. (2012)
*LrSV2*	3BL	Xgwm389, Xgwm533, Xgwm493		Ingala et al. (2012)
*LrG6*	2BL	Xgwm526		Ingala et al. (2012)
*LrFun*	7BL	Xgwm344		Xing et al. (2014)
*LrNJ97*	1BL	Xwmc317 - Xbarc159		Zhao et al. (2013)
*Lr5R*	3DL	Xbarc71 - OPJ-09		Wang et al. (2014)
*LrAc*	5DS	Ta5DS_2737450		Toor et al. (2016)
*LrZH22*	2BS	Xgwm374		Wang et al. (2016)
*LrE1*	7BL	Xgwm131		
*LrP*	5DS	BS00163889		Narang et al. (2019)
*Lr.ace-4A*	4AS	IWA232, IWA1793		Aoun et al. (2019)
*LrM*	2AS	SNP_AX948171722AS		Rani et al. (2020)

## QTLs/MTAs Linked to Lr Genes

In recent years, a number of newer approaches (based on DNA markers) led to the discovery of a large number of QTLs/QRLs and marker-trait associations (MTAs) for resistance against plant diseases including leaf rust. Qualitative resistance provided by Lr genes is generally compromised within a short period of time (Goyeau et al., 2006; Goyeau and Lannou, 2011), but quantitative disease resistance (QDR) provides effective and durable resistance involving major reduction in the level of disease (Mundt et al., 2002; Parlevliet, 2002; Stuthman et al., 2007). The QDR generally depends upon the presence of few major QTLs/genes and a fairly large number of minor QTLs (Ballini et al., 2008; Clair, 2010). Only a solitary example, where QDR for leaf rust resistance has been utilized is the French wheat cultivar Apache, which carried sustained resistance against leaf rust for a fairly long time (Papaïx et al., 2011). The availability of large number of QTLs/MTAs in wheat, as demonstrated in several studies, suggests that QDR against leaf rust is common in this crop, but has not been fully exploited.

A large number of QTLs, mostly associated with Lr genes were listed in some earlier reviews. For instance, in one report, 250 QTLs (reported till 2017) were listed, which were reported in 70 different studies (Pinto da silva et al., 2018). In second study, 35 meta-QTLs (MQTLs) were listed, which were identified using QTLs reported in several studies (Soriano and Royo, 2015). During the last 4 years (after 2017), additional 103 QTLs were reported in 18 studies; 29 of these QTLs were shown to be associated with Lr genes and Lr/Yr genes (Supplementary Table S1).

In addition to QTLs, ∼200 MTAs based on GWAS involving seven association panels (AM) were also reported earlier (Pinto da silva et al., 2018). As mentioned earlier, after publication of this review, ∼600 MTAs were reported in eight genome-wide association studies (GWAS); 42 of these MTAs were found to be linked to Lr genes (Supplementary Table S2). The maximum number of QTLs and Lr genes for leaf rust resistance are present in the B sub-genome. The PVE of the individual QTLs ranged from 4.63% to 75.3%; 29 of these QTLs had a PVE >20% suggesting their utility in MAS for breeding (Supplementary Table S1).

## Wild Relatives as a Source of Lr Genes

At least 50% of Lr genes are derived from wild relatives (alien resources). One of the important alien sources from Fertile Crescent region is Sharon goatgrass (*Aegilops sharonensis*), which is a very valuable source of unique genes/QTLs for resistance to several wheat diseases including leaf rust (for reviews see Ghimire et al., 2020; Figlan et al., 2020). Following other important wild relatives of wheat have also been identified as sources of Lr genes/QTLs: (i) Tausch’s goatgrass (*Ae. tauschii*) (*Lr21*, *Lr22a*, and *Lr39*), (ii) wheatgrass (*Thinopyrum ponticum*) (*Lr24*), (iii) *Ae. geniculate* (*Lr57*), (iv) *Ae. ventricosa* (*Lr37*/*Yr17*), (v) *Ae. umbellulata* (*Lr9*), (vi) *Thinopyrum elongatum* Zhuk. (*Lr 19*), (vii) *Agropyron elongatum* (*Lr24*), (viii) *Secale cereale* L. (*Lr26*), (ix) *Ae. peregrina* (*Lr59*), (x) *Ae. kotschyi (Lr54)*, (xi) *Ae. sharonensis (Lr56)*, (xii) *Ae. triuncialis (Lr58)*, and (xiii) *Ae. neglecta (Lr62)*; however this list is not exclusive (McIntosh, 1975; Autrique et al., 1995; McIntosh et al., 1995; Marais and Botes, 2003; Kumar et al., 2022).

## MAS for Pre-Breeding

**FIGURE 1:**
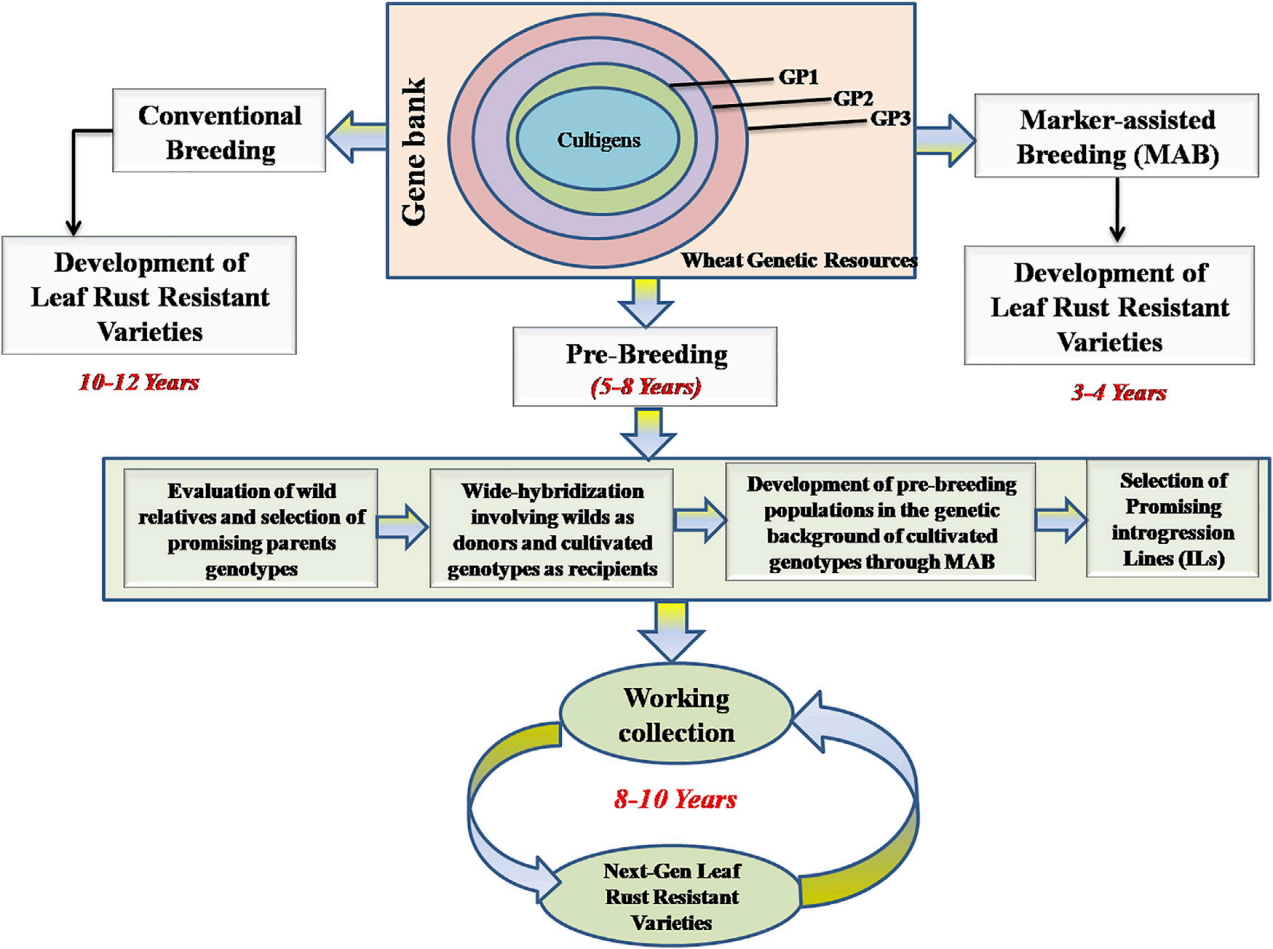
Various pre—breeding steps involved in use of wild relatives in the development of leaf rust resistant wheat varieties. The panels show how wheat genetic resources including wild relatives belonging to primary (GP1), secondary (GP2) and tertiary (GP3) gene pools conserved in different gene banks can be used in pre-breeding programs. The panels also shows the advantages of use of marker—assisted breeding (only 3–4 years in developing new cultivars) over conventional breeding (taking 10–12 years in cultivar development).

There are ∼700 cultivars/varieties from 18 different countries (including India), each cultivar carrying one to six resistance genes for leaf rust including both ASR and APR genes (the details of varieties and their country of origin, are available in Supplementary Tables S2, S4). Two different approaches (including conventional breeding and marker assisted breeding, including pre-breeding) are available for developing resistant cultivars (Figure 1). Since markers associated with each of a number of Lr genes and QTLs including MTAs are available, MAS has become routine for supplementing conventional breeding (Supplementary Table S5). These markers are particularly useful for pyramiding of resistance genes, since introgression of additional resistance genes in the presence of existing resistance genes using phenotypic selection is rather difficult. There are at least a dozen examples (seven from India involving PBW343 and HD2329), where associated markers have been used to supplement conventional breeding including pre-breeding. A number of wheat varieties belonging to hard red winter or soft red winter wheats from United States were also developed using MAS (USDA website; https://www.infoteca.cnptia.embrapa.br/infoteca/bitstream/doc/1124692/1/Doc188-online-Sandra-Brammer.pdf. Using MAS, up to 10 Lr genes could be pyramided into the same wheat cultivar.

## Conclusion and Future Perspectives

The present mini-review is yet another effort to provide a summary of updated published literature on resistance against leaf rust in wheat, including known R genes (∼80 Lr genes and 14 novel genes) (Supplementary Table S1), known QTLs/MTAs (Supplementary Tables S1, S2) and details of varieties containing one or more of these important leaf rust genes/QTLs/MTAs (Supplementary Tables S3, S4). Some details about the use of MAS for introgression of Lr genes into wheat varieties are also included (Supplementary Table S5).

One of the major challenges for wheat breeders is the regular development of new cultivars or improvement of old cultivars using new resistance genes, since new virulence pathotypes and races keep on appearing (Figlan et al., 2020; Ghimire et al., 2020). Therefore, continuous rigorous efforts are needed to locate sources for novel genes/QTLs to overcome new emerging races of pathogen and gain long-term resistance in the field. There are several other areas, which need attention and will certainly be the subject for future research. These will be briefly discussed as the future perspectives.

Although most R genes encode NLR proteins (with NBS-LRR domain), there are several other mechanisms involved as shown in a recent review, where work done during last 25 years involving >300 cloned R genes is reviewed. At least 60% of these R genes were shown to encode NLR proteins, the remaining 40% encoding RLKs/RLPs (Kourelis and van der Hoorn, 2018). Based on the study of these cloned R genes and the corresponding Avr genes of the pathogens, nine different mechanisms for the function of R genes have also been identified and summarised (Kourelis and van der Hoorn, 2018). However, the resistance mechanism of reported Lr genes is not clear and therefore can be a subject for future research.

The most common product of R genes, the NLRs have recently been shown to function through an assembly of a high-resolution structure called ‘resistosome’ which was first resolved in Arabidopsis and is responsible for providing resistance (Wang et al., 2019). Two additional examples of the high-resolution structures of interaction between NLRs and the effector molecules, through formation of resistosome also became available, thus suggesting that formation of the resistosome may be of wide occurrence (Ma et al., 2020; Martin et al., 2020). These three recent studies improved our understanding of the action of NLR at the molecular level. However, no Lr gene has been subjected to such studies involving formation of a resistosome. Therefore this is also an important area of future research.

Another important challenge in breeding for leaf rust resistance is the limited number of Lr genes that have been cloned (*Lr1*, *Lr10, Lr21, Lr22a, Lr34, Lr67*) and therefore cloning more genes is needed to understand the variation between such a large number of Lr genes and the mechanism used for their operation for providing resistance (Dinh et al., 2020; Prasad et al., 2020). According to some optimistic views, it will be possible in the next 15 years to clone most of the ∼460 known wheat resistance genes and their corresponding effectors, making it possible to design suitable strategies for resistance breeding in wheat (Wulff and Krattinger 2022). We, however feel that cloning of so many genes in a short period of 15 years may not be immediately possible. Therefore, closely linked markers may be used to identify which of the Lr genes encode NLR proteins and which other proteins may be encoded by other Lr genes. Bioinformatics may be used for this purpose and the results of this exercise may then be verified using suitable experiments.

Genomics of the pathogen is another important area, since genomes of a number of races of the pathogen have already been sequenced (Kiran et al., 2016; Wu et al., 2020; Fellers et al., 2021). This should facilitate use of bioinformatics for identification of effectors, using knowledge about conserved domains that have been discovered to be present in effector molecules. The genome sequences of different races of Pt have been worked out and many more genomes from the pathogen will also allow us to know the pangenome of Pt, which includes core genome, dispensible genome and unique genome. This knowledge will also allow to identify effectors and in planning suitable strategies for wheat breeding involving resistance against leaf rust.

It may also be necessary to study the effect of environment on expression of many resistance genes in the host since expression of genes has been found to vary with changing temperature (Figlan et al., 2020). This will involve study of the mode of action of resistance genes in the host, their interactions with other host genes, interactions with *Avr* gene while providing stable and durable resistance across environments. The recent advances in genomics tools and techniques including whole genome sequencing, genome annotation and high-throughput genomics tools like pathogenomics, gene cloning, genome editing are expected to offer deeper insights into host-pathogen interactions. This should eventually help in achieving durable rust resistance (Dinh et al., 2020). Molecular biology tools including HIGS/VIGS have also become very important for understanding and analyzing different facets of host and pathogen biology that includes secretome analysis, transcriptional profiling, putative virulence gene identification, structural gene annotation, and alternative transcript splicing. Another important area of future research is identification of vir genes, and effectors, which together make the subject of effectoromics and effector based breeding. This will allow the use of knowledge about effectors to screen the germplasm for resistance.

Epigenomics is another area, which has started attracting the attention of wheat geneticists. This will allow us to understand the role of DNA methylation, histone modifications, noncoding RNAs (e.g., miRNAs, lncRNAs) and chromatin states, thus further resolving the mechanism of resistance at the molecular level (Saripalli et al., 2020a; Saripalli et al., 2020b; Jain et al., 2020; Prasad et al., 2020).
