# Supplementary material for: An Update on Resistance Genes and Their Use in the Development of Leaf Rust Resistant Cultivars in Wheat
Source: Front Genet. 2022 Mar 31;13:816057. doi: 10.3389/fgene.2022.816057 (PMC9008719; doi:10.3389/fgene.2022.816057)
Supplement: Supplementary file 1 [file DataSheet1.docx]

**ESM Table 1** List of QTLs associated with the known Lr genes (Year 2018 onwards) identified using interval mapping.

| QTL (R^2^%) | Chromsome | Total QTL/cross (type of population) | Associated Lr genes | Reference |
| --- | --- | --- | --- | --- |
| *QLr.cim-1BL* (69) | 1BL | 4/Avocet-YrA × Chilero (RIL) | *Lr46/Yr29* | Ponce-Molina et al., 2018 |
| *QLr.hebau-7DS* (20.9) | 7DS | 8/Zhou 8425B x Chinese Spring (RIL) | *Lr34* | Zhang et al., 2017 |
| *QLr.hebau-1BL* (18.9) | 1BL | 5/Fuyu 3×Zhengzhou 5389 (RIL) | *Lr46/Yr29* | Gebrewahidet al., 2019a |
| *QLR.IPBB-2B* (18) | 2B | 10/PamyatiAzieva × Paragon (RIL) | *Lr35, Lr50* | Genievskayaet al., 2019 |
| *QLR.IPBB-3B.1* (27) | 3B |  | *Lr27, Lr74* |  |
| *QLr.crc-1BL* (16.61) | 1BL | 6/Toropi-6.4 × Thatcher (DH) | *Lr46/Yr29* | Rosa et al., 2019 |
| *QLr.crc-5DS* (4.63) | 5DS |  | *Lr78* |  |
| *QLr.hebau-1BL/QYr.hebau-1BL* (12.6) | 1BL | 7/SW 8588 × Thatcher (RIL) | *Lr46/Yr29* | Zhang et al.,2019 |
| *QLr.spa-1A* (8.6) | 1A | 13/Carberry x AC Cadillac (DH) | *Lr10* | Bokoreet al., 2020 |
| *QLr.spa-2B.1* (8.7) | 2B |  | Lr16 |  |
| *QLr.sun-1BL* (22) | 1BL | 3/Aus27506 x Aus27229 (RIL) | Lr46 | Kandiahet al., 2020 |
| *QLr.uga.2BS* (75.3) | 2BS | 1/AGS 2000 x 26R61 (RIL) | *LrA2k* | Sapkotaet al., 2019 |
| *QYr.hbau-1BL/QLr.hbau-1BL* (12.7) | 1BL | 4/Mianyang351-15/Zhengzhou 5389 | *Lr46/Yr29* | Gebrewahidet al., 2019b |
| *QYr.hbau-2AS/QLr.hbau-2AS* (21.3) | 2AS |  | *Lr37* |  |
| *QYr.hbau-7BL/QLr.hbau-7BL* (15) | 7BL |  | *Lr68* |  |
| *QLr.cim-2BC* (26.2) | 2B | 4/Atred#2 × Dunkler , 4/Atred#2 × Heller#1 (RIL) | *Lr46* | Li et al., 2019 |
| *QLr.cim-1BL.1/QYr.cim-1BL.1* (51.1) | 1BL | 4/Apav#1 x Arableu#1 | *Lr46/Yr29* | Yuan et al., 2020 |
| *QLr.cim-7BL* (31.3) | 7BL |  | *Lr68* |  |
| *QLr.usw-1BL.2* (7.1) | 1BL | 3/Saragolla/ATRED #2 | *Lr46* | Kthiriet al., 2019 |
| *QLr.usw-1BL.1* (17.7) | 1BL | 2/Arnacoris/ATRED #2 | *Lr46* |  |

**ESM Table 2** MTAs and QTLs worked out based on LD that are associated with known Lr genes identified using GWAS (Year 2018 onwards).

| QTLs^*^/MTA associated to Lr gene (Total MTAs) | No. of genotypes/Associated genes |
| --- | --- |
| QTL^*^ (marker interval) | |
| Muhammad et al., 2018 | |
| *QLr.uaf.1AS* (*gwm136-gwm33*)(42) | *225/2Lr10* |
| *QLr.uaf.1BL .2* (*wmc419-gpw4002*) | *225/Lr33* |
| *QLr.uaf.1DS* (*gwm354-cfd15*) | *225/Lr10* |
| *QLr.uaf.2AS* (*barc124-gwm497*) | *225/Lr17a* |
| *QLr.uaf.2BS* (*gwm219-wmc661*) | *225/Lr16* |
| *QLr.uaf.2DS* (*gwm210-wmc453*) | *225/Lr39, Lr41* |
| *QLr.uaf.3BS* (*barc75-gwm533*) | *225/LrSV2, Lr27* |
| *QLr.uaf.3DS* (*gwm114-wmc674*) | *225/LrSV2, Lr27* |
| *QLr.uaf.4BS* (*gwm538-gwm368*) | *225/Lr25, 225/Lr49, Lr12* |
| *QLr.uaf.5BS* (*gwm443-sun199*) | *225/Lr52* |
| *QLr.uaf.5DS* (*cfd81-gwm358*) | *225/LrAC* |
| *QLr.uaf.5DL* (*gwm469-gwm465*) | *225/Lr1* |
| *QLr.uaf.6BL* (*gwm626-wmc621*) | *225/Lr3, Lr9* |
| *QLr.uaf.6DS* (*barc183-gwm469*) | *225/Lr53* |
| *QLr.uaf.7AS* (*gwm233-gwm60*) | *225/Lr47* |
| *QLr.uaf.7BS.1* (*Wmc606*) | *225/Lr72* |
| *QLr.uaf.7DS* (*cslv34-gwm295*) | *225/Lr34* |
| MTAs | |
| Sapkotaet al., 2019 | |
| *IWA8221* (32) | *297/LrA2K* |
| Kumar et al., 2020 | |
| *AX-95147877, AX-94411794, AX95014734* (151) | *483/ Lr64* |
| *AX-94674448* | *483/Lr34/Yr18/Sr57* |
| Joukhadaret al., 2020 | |
| *65326_2B* (161) | *2300/Lr48, Lr23,* |
| *40680_3D* | *2300/Lr24/Sr24* |
| *67745_4A* | *2300/Lr28* |
| *33694_6B* | *2300/Lr3* |

^*^For converting MTAs into QTL, LD information was utilised. The total number of MTAs are indicated only once for each study.

**ESM Table 3:** Number of wheat varieties from different countries containing different Lr genes for leaf rust resistance.

| Gene | No. of varieties | Resistance type | Reference |
| --- | --- | --- | --- |
| *Lr*unknown | 1 | - | Bhardwaj et al., 2019 |
| *Lr1* | 4 | SR^*^ | Khan et al., 2017; Li et al., 2018; Zhang et al., 2019; Gupta et al., 2018 |
| *Lr10* | 6 | SR | Gul’tyaevaet al., 2009; Drazet al., 2015; Roelfset al., 1992; Li et al., 2018; Zhang et al., 2019; Pathanet al., 2006; Gupta et al., 2018 |
| *Lr11* | 2 | SR | Roelfset al., 1992; Zhang et al., 2019 |
| *Lr13* | 45 | APR^**^, SR | Bhardwaj et al., 2010; Malakeret al., 2011; Cuddy et al., 2016; Tomeret al., 2014; Khan et al., 2017; Roelfset al.,1992; Sivasamy 2014; Zhang et al., 2019; Pathanet al., 2006 |
| *Lr14a* | 5 | SR | Tomeret al., 2014; Khan et al., 2017; Zhang et al., 2019; Pathanet al., 2006 |
| *Lr14b* | 1 | SR | Zhang et al., 2019 |
| *Lr15* | 2 | SR | Zhang et al., 2019; Gupta et al., 2018 |
| *Lr16* | 1 | SR | Zhang et al., 2019 |
| *Lr17* | 1 | SR | Zhang et al., 2019 |
| *Lr17a* | 2 | SR | Cuddy et al., 2016; Gupta et al., 2018 |
| *Lr18* | 3 | SR | Drazet al., 2015; Li et al., 2018; Zhang et al., 2019 |
| *Lr19* | 16 | APR, SR | Drazet al., 2015; Gul’tyaevaet al., 2009; Tomeret al., 2014; Sivasamy 2014; Zhang et al., 2019; Bhardwaj et al., 2019 |
| *Lr20* | 13 | APR | Zhang et al., 2019; Pathanet al., 2006; Mebrateet al., 2008 |
| *Lr21* | 3 | APR | Drazet al., 2015; Zhang et al., 2019; Mebrateet al., 2008 |
| *Lr22b* | 1 | SR | Roelfset al., 1992 |
| *Lr23* | 10 | SR | Bhardwaj et al., 2010; Tomeret al., 2014; Khan et al., 2017; Sivasamy 2014; Zhang et al., 2019 |
| *Lr24* | 45 | APR, SR | Drazet al., 2015; Tomeret al., 2014; Gul’tyaevaet al., 2009; Cuddy et al., 2016; Khan et al., 2017; Sivasamy 2014 ; Zhang et al., 2019; Bhardwaj et al., 2019; Gupta et al., 2018 |
| *Lr26* | 15 | SR | Cuddy et al., 2016; Li et al., 2018; Zhang et al., 2019; Drazet al., 2015; Bhardwaj et al., 2010; Pathanet al., 2006 |
| *Lr28* | 9 | SR | Bhardwaj et al., 2019; Tomeret al., 2014; Sivasamy 2014; Zhang et al., 2019 |
| *Lr29* | 2 | SR, APR | Drazet al., 2015; Zhang et al., 2019 |
| *Lr2a* | 2 | SR | Zhang et al., 2019; Gupta et al., 2018 |
| *Lr2b* | 1 | SR | Zhang et al., 2019 |
| *Lr2c* | 3 | APR, SR | Drazet al., 2015; Zhang et al., 2019; Gupta et al., 2018 |
| *Lr3* | 8 | SR | Li et al., 2018; Zhang et al., 2019; Mebrateet al., 2008 |
| *Lr30* | 1 | APR | Zhang et al., 2019 |
| *Lr32* | 3 | SR, APR | Li et al., 2018; Sivasamy 2014 |
| *Lr33* | 1 | SR | Zhang et al., 2019 |
| *Lr34* | 19 | APR, SR | Gupta et al., 2018; Sivasamy 2014 ; Singh and Rajaram 2002; Zhang et al., 2019 |
| *Lr35* | 3 | APR | Sivasamy 2014 |
| *Lr36* | 1 | SR | Zhang et al., 2019 |
| *Lr37* | 5 | APR | Cuddy et al., 2016; Drazet al., 2015 |
| *Lr3a* | 4 | SR | Cuddy et al., 2016; Pathanet al., 2006; Gupta et al., 2018 |
| *Lr3bg* | 1 | SR | Zhang et al., 2019 |
| *Lr3ka* | 2 | SR | Zhang et al., 2019; Pathanet al., 2006 |
| *Lr44* | 2 | APR | Zhang et al., 2019; Mebrateet al., 2008 |
| *Lr45* | 4 | SR | Sivasamy 2014; Zhang et al., 2019 |
| *Lr47* | 1 | ^***^ASR | Zhang et al., 2019 |
| *Lr51* | 1 | ASR | Zhang et al., 2019 |
| *Lr53* | 1 | ASR | Zhang et al., 2019 |
| *Lr9* | 16 | SR, APR | Drazet al., 2015; El-Orabeyet al., 2013; Gul’tyaevaet al., 2009; Sivasamy 2014; Zhang et al., 2019; Mebrateet al., 2008; Gupta et al., 2018 |
| *LrB* | 1 | - | Zhang et al., 2019 |
| *LrU* | 1 | - | Gupta et al., 2018 |
| *Lr34, Lr36* | 1 | APR, SR | El-Orabeyet al., 2013 |
| *Lr1, Lr26* | 9 | SR | Bhardwaj et al., 2010; Malakeret al., 2011; Li et al., 2018; Khan et al., 2017 |
| *Lr1, Lr23* | 2 | SR | Bhardwaj et al., 2010; Khan et al., 2017 |
| *Lr1, Lr24* | 1 | SR, APR | Cuddy et al., 2016 |
| *Lr1, Lr3* | 2 | SR | Bhardwaj et al., 2010; Li et al., 2018 |
| *Lr1, Lr37* | 5 | APR, SR | Cuddy et al., 2016 |
| *Lr10, Lr13* | 20 | APR, SR | Malakeret al., 2011; Khan et al., 2017; Sivasamy 2014; Pathanet al., 2006 |
| *Lr10, Lr23* | 2 | SR | Khan et al., 2017; Sivasamy 2014 |
| *Lr10, Lr37* | 2 | - |  |
| *Lr1, Lr13* | 9 | SR, APR | Bhardwaj et al., 2010; Malakeret al., 2011; Cuddy et al., 2016; Khan et al.,2017 |
| *Lr13, Lr14a* | 3 | SR | Pathanet al., 2006 |
| *Lr13, Lr17* | 1 | SR | Roelfset al., 1992 |
| *Lr13, Lr17b* | 4 |  | Pathanet al., 2006 |
| *Lr13, Lr20* | 3 | SR | Cuddy et al., 2016 |
| *Lr13, Lr23* | 2 | APR, SR | Malakeret al., 2011 |
| *Lr13, Lr24* | 1 | SR, APR | Cuddy et al., 2016 |
| *Lr13, Lr26* | 2 |  | Pathanet al., 2006 |
| *Lr3, Lr13* | 1 | APR, SR | Bhardwaj et al., 2010 |
| *Lr13, Lr34* | 4 | APR, SR | Bhardwaj et al., 2010; Roelfset al., 1992 |
| *Lr13, Lr37* | 7 | APR, SR | El-Orabeyet al., 2013; Cuddy et al., 2016; Pathanet al., 2006 |
| *Lr13, Lr41* | 1 | APR, SR | El-Orabeyet al., 2013 |
| *Lr13, UGIR* | 1 | SR | Pathanet al., 2006 |
| *Lr14a, Lr20* | 1 | - | Pathanet al., 2006 |
| *Lr14a, Lr35* | 1 | SR, APR | Tomeret al., 2014 |
| *Lr14a, Lr37* | 1 | SR | Pathanet al., 2006 |
| *Lr16, Lr13* | 1 | SR | Kolmeret al., 2002 |
| *Lr20, Lr26* | 1 | SR | Pathanet al., 2006 |
| *Lr20, Lr37* | 1 | SR, APR | Cuddy et al., 2016 |
| *Lr23, Lr26* | 10 | SR | Bhardwaj et al., 2010; Sivasamy 2014; Khan et al., 2017 |
| *Lr23, Lr34* | 4 | APR, SR | Bhardwaj et al., 2010; Tomeret al., 2014; Sivasamy 2014 |
| *Lr23, Lr46* | 1 | SR | Cuddy et al., 2016 |
| *Lr24, Lr28* | 1 | - | Gupta et al., 2018 |
| *Lr24, Lr37* | 4 | APR, SR | Cuddy et al., 2016 |
| *Lr26, Lr34* | 4 | APR, SR | Bhardwaj et al., 2010; Sivasamy 2014 |
| *Lr26, Lr37* | 1 | - | Pathanet al., 2006 |
| *Lr3, Lr26* | 6 | SR | Malakeret al., 2011; Li et al., 2018; Khan et al., 2017 |
| *Lr35, Lr26* | 1 | SR | Sivasamy 2014 |
| *Lr3a, Lr13* | 4 | - | Pathanet al., 2006 |
| *Lr3a, Lr14a* | 1 | - | Pathanet al., 2006 |
| *Lr3a, Lr17a* | 1 | SR | Cuddy et al., 2016 |
| *Lr3a, Lr23* | 2 | SR | Cuddy et al., 2016 |
| *Lr3a, Lr37* | 3 | APR, SR | Cuddy et al., 2016 |
| *Lr9, Lr10* | 1 | SR | Gul’tyaevaet al., 2009 |
| *Lr1, Lr13, Lr37* | 2 | APR, SR | Cuddy et al., 2016 |
| *Lr1,Lr20, Lr36* | 1 | SR | Zhang et al., 2019 |
| *Lr1, Lr23, Lr26* | 5 | SR | Bhardwaj et al., 2010; Sivasamy 2014; Khan et al., 2017 |
| *Lr1, Lr24, Lr37* | 1 | APR, SR | Cuddy et al., 2016 |
| *Lr1, Lr26, Lr24* | 1 | APR, SR | Khan et al., 2017 |
| *Lr1, Lr26, Lr34* | 3 | APR, SR | Malakeret al., 2011; Sivasamy 2014 |
| *Lr1, Lr26, Lr37* | 1 | - | Pathanet al., 2006 |
| *Lr1, Lr3a or Lr73, Lr13* | 1 | APR, SR | Cuddy et al., 2016 |
| *Lr1, Lr3a, Lr13* | 1 | - | Pathanet al., 2006 |
| *Lr10, Lr13, Lr14a* | 1 | - | Pathanet al., 2006 |
| *Lr10, Lr13, Lr26* | 4 | - | Pathanet al., 2006 |
| *Lr10, Lr13, Lr34* | 1 | APR, SR | Bhardwaj et al., 2010 |
| *Lr10, Lr13, Lr37* | 1 | - | Pathanet al., 2006 |
| *Lr13, Lr16, Lr34* | 1 | APR, SR | Kolmeret al., 2002 |
| *Lr13, Lr17b, Lr37* | 1 | - | Pathanet al., 2006 |
| *Lr13, Lr23, Lr34* | 4 | - | Tomeret al., 2014 |
| *Lr13, Lr24, Lr34* | 1 | APR, SR | El-Orabeyet al., 2013 |
| *Lr13, Lr24, Lr35* | 1 | APR, SR | El-Orabeyet al., 2013 |
| *Lr13, Lr26, Lr37* | 1 | - | Pathanet al., 2006 |
| *Lr13, Lr27, Lr31* | 1 | SR, APR | Cuddy et al., 2016 |
| *Lr13, Lr34, Lr35* | 1 | APR, SR | Sivasamy 2014 |
| *Lr14a, Lr17, Lr34* | 2 | APR, SR | Roelfset al., 1992 |
| *Lr17, Lr26, Lr32* | 1 | SR | Li et al., 2018 |
| *Lr23, Lr26, Lr34* | 1 | APR, SR | Sivasamy 2014 |
| *Lr23, Lr26, Lr37* | 1 | SR, APR | Cuddy et al., 2016 |
| *Lr26, Lr34, Lr35* | 1 | APR, SR | Bhardwaj et al., 2010 |
| *Lr27, Lr31, Lr37* | 1 | SR, APR | Cuddy et al., 2016 |
| *Lr2b, Lr14a,Lr21* | 1 | SR | Zhang et al., 2019 |
| *Lr3, Lr10, Lr13* | 2 | APR, SR | Khan et al., 2017 |
| *Lr34, Lr46, Lr68* | 1 | APR, SR | Tomeret al., 2014 |
| *Lr3a or Lr23, Lr27, Lr31* | 1 | APR, SR | Cuddy et al., 2016 |
| *Lr3a, Lr13, Lr26* | 5 | - | Pathanet al., 2006 |
| *Lr3a, Lr26, Lr37* | 1 | - | Pathanet al., 2006 |
| *LrTb, Lr13, Lr16* | 11 | SR | Kolmeret al., 2002 |
| *Lr1, Lr3, Lr26, Lr32* | 1 | SR | Li et al., 2018 |
| *Lr1, Lr13, Lr10, Lr34* | 1 | APR, SR | Bhardwaj et al., 2010 |
| *Lr1, Lr27, Lr31, Lr37* | 3 | SR, APR | Cuddy et al., 2016 |
| *Lr12, Lr13, Lr34, Lr36* | 11 | APR, SR | El-Orabeyet al., 2013 |
| *Lr13, Lr17, Lr23, Lr34* | 1 | APR, SR | Tomeret al., 2014 |
| *Lr13, Lr34, Lr35, Lr36* | 1 | APR, SR | El-Orabeyet al., 2013 |
| *Lr9, Lr12, Lr13, Lr24, Lr34* | 1 | APR, SR | El-Orabeyet al., 2013 |
| *Lr9, Lr13, Lr24, Lr34, Lr41* | 11 | APR, SR | El-Orabeyet al., 2013 |
| *Lr1, Lr3, Lr10, Lr13, Lr23, Lr26* | 1 | SR | Tomeret al., 2014 |
| *Lr10, Lr13, Ltn* | 1 | APR, SR | Malakeret al., 2011 |
| *Lr13, Lr20, Lr37, Lr46* | 1 | SR, APR | Cuddy et al., 2016 |
| *Lr19, Lr24, Lr26, Sr24, Sr25, Sr31, Yr9* | 1 | APR, SR | Bhardwaj et al., 2019 |
| *Lr19, Lr26, Sr25, Sr31, Yr9, Yr27* | 11 | APR, SR | Bhardwaj et al., 2019 |
| *Lr24, Lr26, Sr2, Sr24, Sr31, Yr9* | 1 | APR, SR | Bhardwaj et al., 2019 |
| *Lr24, Lr26, Sr24, Sr31, Yr9, Yr27* | 1 | APR, SR | Bhardwaj et al., 2019 |
| *Lr24, Sr24, Sr31* | 1 | APR | Sivasamy 2014 |
| *Lr24, Sr32* | 1 | APR | Bhardwaj et al., 2019 |
| *Lr24, Sr43* | 1 | APR | Bhardwaj et al., 2019 |
| *Lr26, Lr19, Lr24, Sr31, Yr9, YrPBW343* | 11 | APR, SR | Bhardwaj et al., 2019 |
| *Lr26, Lr24, Sr24, Sr31, Yr9, Yr15* | 11 | APR, SR | Bhardwaj et al., 2019 |
| *Lr26, Lr28, Sr31, Yr9, Yr15* | 1 | SR | Bhardwaj et al., 2019 |
| *Lr26, Lr28, Sr31, Yr9, YrCD* | 1 | SR | Bhardwaj et al., 2019 |
| *Lr26, Lr28, Sr31, Yr9, YrChina-84* | 1 | SR | Bhardwaj et al., 2019 |
| *Lr26, Lr32, Sr31, Yr9, YrPBW343* | 1 | APR, SR | Bhardwaj et al., 2019 |
| *Lr26, Lr39, Sr31, Yr9, Yr (PBW343)* | 1 | SR | Bhardwaj et al., 2019 |
| *Lr26, Sr31, Yr9, Yr10* | 1 | SR | Bhardwaj et al., 2019 |
| *Lr26, Sr31, Yr9, Yr5* | 1 | SR | Bhardwaj et al., 2019 |
| *Lr26, Sr31, Yr9, YrChina-84* | 1 | SR | Bhardwaj et al., 2019 |
| *Lr28, Lr26, Sr31, Yr9, Yr27* | 1 | SR | Bhardwaj et al., 2019 |
| *Lr42, Lr26, Sr31, Yr9, Yr27* | 1 | SR | Bhardwaj et al., 2019 |
| *Lr45, Lr26, Sr31, Yr9, Yr27* | 1 | SR | Bhardwaj et al., 2019 |
| *Lr46, Yr29* | 1 | APR | Tomeret al., 2014 |
| *Lr57, Yr40* | 2 |  | Gupta et al., 2018 |
| *Lr57/Yr40, Lr37/Yr17* | 1 | APR, SR | Gupta et al., 2018 |
| *Lr67, Yr46* | 1 | APR | Tomeret al., 2014 |
| *Lr9, Lr24, Sr2, Sr24* | 1 | APR, SR | Bhardwaj et al., 2019 |

^*^SR: Seedling resistance, ^**^APR:Adult plant resistance; ^***^ASR: All stage resistance.

**ESM Table 4.** Leaf rust resistant wheat varieties from India (I), China (Ch), Egypt (E), USA, UK , Germany (G), France (F), Switzerland (S), Czech Republic (CR), Italy (IT), Hungary (H), Holland (Ho), Ethopia (Et), Canada (C), Australia (A), Bangladesh (B) and Russia (R)

| Variety | Gene | Resistance Type | Reference |
| --- | --- | --- | --- |
| FKW3 (06005) (I) | *Lr unknown* |  | Bhardwaj et al 2019 |
| HS 507 (I), Yannong 15 (Ch), RL6003 (Ch), HI KK1 (I) | *Lr1* | SR^*^ | Khan et al 2014; Li et al 2018; Zhang et al 2019; Gupta et al 2017 |
| Omskaya 32 (R), Omskaya 35 (R), Skala (R), Simbirtsit (R), Saratovskaya 29 (R), Saratovskaya 60 (R), Tuleevskaya (R), Strada Sibiri (R), Tulundskaya 12 (R), Khabarovchanka (R), Eskada 6 (R), Eskada 70 (R), Erythrospermum 59 (R), Yugo-Vostochnaya 2 (R), Yugo-Vostochnaya 4 (R), Altaiskaya 325 (R), Altaiskaya 60 (R), Zhnitsa (R), Karagandinskaya 70 (R), Izumrudnaya (R), Novosibirskaya 89 (R), Omskaya 20 (R), Omskaya 29 (R), Obskaya 14 (R), Omskaya 12 (R), Bashkirskaya 26 (R), Leningradka (R), Novosibirskaya 29 (R), Novosibirskaya 15 (R), Lyuba (R), L-503 (R), Krest’yanka (R), Kerba (R), Voronezhskaya 12 (R), Al’bidum 31 (R), Voronezhskaya 10 (R), Bagaevskaya 93 (R), Amir (R), Al’bidum 29 (R), Al’bidum 23 (R), Vetluzhanka, (R), Prokhorovka (R), Primorskaya 40 (R), Rostovchanka 5 (R), Noga (R), Del’ta (R), Zimorodok (R), Yubileinaya 75 (R), Vita (R), Malakhit (R), Lira (R), Moskovich (R), Zernogradka 8 (R), Kupava (R), Gemmiza9 (E), Lee (USA), Zhouyuan 187 (Ch), RL6004 (Ch), PH01 (Ch), Pegaso (It), HI KK6 (I) | *Lr10* | SR | Gul’tyaeva et al 2009; Draz et al 2015; Roelfs et al ; Li et al 2018; Zhang et al 2019; Pathan et al 2006; Gupta et al 2017 |
| Bulgaria 88 (USA), Hussar (Ch) | *Lr11* | SR | Roelfs et al; Zhang et al 2019 |
| GW 322, VL 824 (I), BARI Gom-25 (B), Hydra (A), Jade (A), Arjun (I), Sonalika (I), Kalyansona (I), Lok 1 (I), HD 3059 (I), HD 3118 (I), MP 3336 (I), WH 1080 (I), WH 1105 (I), Sonalika (USA), HUW 468 (I), K 8027 (I), Manjtou (Ch), Buster (UK), Pegassos (G), Runal (S), Spark (UK), Texel (F), Thesee (F), Transit (G), Cadenza (UK), Capo (Au), David (H), Forby (F), Galaxie (S), Gobe (H), Josef (Au), Kontrast (G), Boka (CR), Lindos (Au), Moulin (UK), MV Magvas (H), Pastiche (UK), Bontaris (G), Achat (Au), Arina (S), Artaban (F), Aztec (F), Batis (G), Orqual (F) | *Lr13* | APR^**^,SR | Bhardwaj et al 2010; Malaker et al 2011; Cuddy et al 2016; Tomer et al 2014; Khan et al 2014; Roelfs et al ; Sivasamy 2014 ; Zhang et al 2019; Pathan et al 2006 |
| WL 711 (A), HI 8713 (d) (I), RL6013(Ch), Bruta (CR), Forno (S) | *Lr14a* | SR | Tomer et al 2014; Khan et al 2014; Zhang et al 2019; Pathan et al 2006 |
| RL6006 (Ch) | *Lr14b* | SR | Zhang et al 2019 |
| RL6052 (Ch), HI KK7 (I) | *Lr15* | SR | Zhang et al 2019; Gupta et al 2017 |
| RL6005 (Ch) | *Lr16* | SR | Zhang et al 2019 |
| RL6008 (Ch) | *Lr17* | SR | Zhang et al 2019 |
| Baxter (A), HI KK8 (I) | *Lr17a* | SR | Cuddy et al 2016; Gupta et al 2017 |
| Gemmiza10 (R), Taishan 269 (Ch), RL6009 (Ch), | *Lr18* | SR | Draz et al 2015; Li et al 2018; Zhang et al 2019 |
| Gemmiza11 (R), Tupaikovskaya 10 (R), Volgoural`skaya (R), Ekada 6 (R),Fora (R), L505 (R), L503 (R), Dobrynya (R), Yuliya (R), HD 2932 (I), HW 2045 (I), HW 2044 (I), HW 2078 (I), RL6040 (Ch), FLW8 (04012) (I), HW2049 (04016) (I) | *Lr19* | APR, SR | Draz et al 2015; Gul’tyaeva et al 2009; Tomer et al 2014; Sivasamy 2014 ; Zhang et al 2019; Bhardwaj et al 2019 |
| RL6092 (Ch), Maris Halberd (UK), Sicco (UK), Szalka (H), Timmo (UK), Sappo (UK), Fasan (Et, G), Epos (Et, G), Quattro (Et, G), HI KK9 (I) | *Lr20* | APR | Zhang et al 2019; Pathan et al 2006; Mebrate et al 2008 |
| Sids12 (E), RL6043 (Ch), Granny (Et, G) | *Lr21* | APR | Draz et al 2015; Zhang et al 2019; Mebrate et al 2008 |
| Thatcher (USA) | *Lr22b* | SR | Roelfs et al |
| A-9-30-1 (I), MACS 2846 (I), HD 2285 (I), HD 3043 (I), HI 8737 (d) (I), NW 5054 (I), TL 2969 (T) (I), UAS 428 (d) (I), HD 2285 (I), RL6012 (Ch) | *Lr23* | SR | Bhardwaj et al 2010; Tomer et al 2014; Khan et al 2014; Sivasamy 2014 ; Zhang et al 2019 |
| Sids13 (E), DL 784-3 (Vidisha) (I), HW 2004 (Amar) (I), DL 788-2 (Vaishali) (I), HW 2045 (Kausambi) (I), HD 2781 (Aditya) (I), HI 1500 (Amrita) (I), MP 4010 (I), Raj 4037 (I), HD 2851(Pusa Vishesh) (I), HD 2833 (Tripti) (I), NW 1067 (I), HI 1531 (I), COW(W)-1 (I), HD 2888 (Pusa Wheat) (I), AKAW 4627 (I), AKAW 3722 (Vimal) (I), Yugo-Vostochnaya 2 (R), Elmore CL Plus (A), Impress CL Plus (A), Janz (A), Lancer (A), Lang (A), Sunguard (A), MP 3288 (I), RAJ 4238 (I), HW 2004 (I), HD 2833 (I), HW 2094 (I), HW 2021 (I), HW 2023 (I), HW 5207/HW 5207-1 (I), CoW(W)1 (I), HW 2095 (I), HW 2025 (I), HW 2091 (I), RL6064 (Ch), FLW1 (03013) (I), FLW5 (03017) (I), HW2002(04014) (I), HW 5216 (PUSA THENMALAI) (I), HW 3070 (I), HW 5001 (I), HD 2888 (PUSA WHEAT 107) (I), | *Lr24* | APR, SR | Draz et al 2015; Tomer et al 2014; Gul’tyaeva et al 2009; Cuddy et al 2016; Khan et al 2014; Sivasamy 2014 ; Zhang et al 2019; Bhardwaj et al 2019; IIWBR Karnal |
| Sentinel (A), Luyuan 301 (Ch), Liao 9629 (Ch), Liao 9638 (Ch), Yan C96 (Ch), RL6078 (Ch), Misr1 (E), K 2008 (I), HS 418 (I), K 9904 (I), PBW 373 (I), K 9904 (I), Csuros (H), Ikarus (Au), MV Magdalena (H) | *Lr26* | SR | Cuddy et al 2016; Li et al 2018; Zhang et al 2019; Draz et al 2015; Bhardwaj et al 2010; Pathan et al 2006 |
| HW 2031(04015) (I), MACS 6145 (I), HW 2034 (I), HW 2088 (I), HW 2096 (I), HW 2099 (I), HW 2024 (I), HW 2093 (I), RL6079 (Ch) | *Lr28* | SR | Bhardwaj et al 2019; Tomer et al 2014; Sivasamy 2014 ; Zhang et al 2019 |
| Misr2 (E), RL6080 (Ch) | *Lr29* | SR, APR | Draz et al 2015; Zhang et al 2019 |
| RL6016 (Ch), HI KK2 (I) | *Lr2a* | SR | Zhang et al 2019; Gupta et al 2017 |
| RL6019 (Ch) | *Lr2b* | SR | Zhang et al 2019 |
| Sakha94 (E), RL6047 (Ch), HI KK3 (I) | *Lr2c* | APR, SR | Draz et al 2015; Zhang et al 2019; Gupta et al 2017 |
| Liangxing 66 (Ch), Bin 02-47 (Ch), Linmai No.4 (Ch), RL6002 (Ch), Dereselign (Et, G), Katar (Et, G) | *Lr3* | SR | Li et al 2018; Zhang et al 2019; Mebrate et al 2008 |
| RL6049 (Ch) | *Lr30* | APR | Zhang et al 2019 |
| Yan 896063 (Ch), HW 2089 (I), HW 2090 (I) | *Lr32* | SR, APR | Li et al 2018; Sivasamy 2014 |
| RL6057 (Ch) | *Lr33* | SR | Zhang et al 2019 |
| HD3086  (Pusa Gautami) (I), C 306 (I), NI 5439 (I), Jupateco 73R (USA), Nacozari 76 (USA), Sonoita 81 (USA), Frontana (USA) Baiguozitou (Ch), Baimazhatou (Ch), Baitutou (Ch), Hongguangtou (Ch), Hongtutou (Ch), Xinlijun (Ch), Zijielumai (Ch), Crow (Ch), Esmeralda 86 (Ch), Mango (Ch), Ocoroni 86 (Ch), Tonichi 81 (Ch), Trap (Ch) | *Lr34* | APR, SR | IIWBR Karnal; Sivasamy 2014 ; R p singh; Zhang et al 2019 |
| Lok -1 (I), PBW 502 (I), HD 2687 (I), | *Lr35* | APR | Sivasamy 2014 |
| E84018 (Ch) | *Lr36* | SR | Zhang et al 2019 |
| Preston (A), Scout (A), Scenario (A), Steel (A), Sunvale (A), Gemmeiza 7 (E), | *Lr37* | APR | Cuddy et al 2016; Draz et al 2015 |
| Waagan (A), Chablis (UK), Viginta (CR), HI KK4 (I) | *Lr3a* | SR | Cuddy et al 2016; Pathan et al 2006; Gupta et al 2017 |
| RL6042 (Ch) | *Lr3bg* | SR | Zhang et al 2019 |
| RL6007 (Ch), Blava (CR) | *Lr3ka* | SR | Zhang et al 2019; Pathan et al 2006 |
| RL6147 (Ch), Kubsa (Et, G) | *Lr44* | APR | Zhang et al 2019; Mebrate et al 2008 |
| HW4231 – 53 (I), HW 4301 - 4323 (I), HW 4261 – 4283 (I), RL 6144 (Ch) | *Lr45* | SR | Sivasamy 2014 ; Zhang et al 2019 |
| C98.006 (Ch) | *Lr47* | ^***^ASR | Zhang et al 2019 |
| C78.5 (Ch) | *Lr51* | ASR | Zhang et al 2019 |
| 98M71 (Ch) | *Lr53* | ASR | Zhang et al 2019 |
| Giza168 (E), Giza 162 (E), Giza 163 (E), Chelyaba 2 (R), Pamyati Ryuba (R), Splav (R), Nemchinovskaya 24 (R), HP 1633 (I), RL6010 (Ch), Bobitcho (Et, G), Tussie (Et, G), Tybalt (Et, G), HI KK5 (I) | *Lr9* | SR, APR | Draz et al 2015; El-Orabey et al 2013; Gul’tyaeva et al 2009; Sivasamy 2014 ; Zhang et al 2019; Mebrate et al 2008; Gupta et al 2017 |
| RL6051 (Ch) | *LrB* | - | Zhang et al 2019 |
| PAU 16057 (I) | *LrU* | - | Gupta et al 2017 |
| Sakha 61 (E) | *Lr34, Lr36* | APR, SR | El-Orabey et al 2013 |
| HD 2808 (I), SKW 191 (I), HS 365 (I), Gourab (B), Zemai No.1 (Ch), Weimai No.8 (Ch), Prodip (B), HW 5216 (I), | *Lr1, Lr26* | SR | Bhardwaj et al 2010; Malaker et al 2011; Li et al 2018; Khan et al 2014 |
| K 9943 (I), MACS 6478 (I), | *Lr1, Lr23* | SR | Bhardwaj et al 2010; Khan et al 2014 |
| Magenta (A) | *Lr1, Lr24* | SR, APR | Cuddy et al 2016 |
| KRL 35 (I), Liao 9518 (Ch) | *Lr1, Lr3* | SR | Bhardwaj et al 2010; Li et al 2018 |
| Envoy (A), Estoc (A), Hatchet CL Plus (A), Harper (A), Kord CL Plus (A) | *Lr1, Lr37* | APR, SR | Cuddy et al 2016 |
| Akbar (B), Aghrani (B) DBW 88 (I), DBW 90 (I), DPW 621–50 (I), HPW 349 (I), HS 542 (I), UAS 347 (I),WH 1124 (I), GW 273 (I), RAJ 3765 (I), Tremie (Ho, F), Charger (UK), Consort (UK), Danis (S), Greif (G), Hereward (UK, Ho), Mercia (UK), Ortop (F) | *Lr10, Lr13* | APR, SR | Malaker et al 2011; Khan et al 2014; Sivasamy 2014 ; Pathan et al 2006 |
| K 1006 (I), HDR 77 (I) | *Lr10, Lr23* | SR | Khan et al 2014; Sivasamy 2014 |
| Terza (S) | *Lr10, Lr37* |  |  |
| K 9644 (I), Ananda (B), Shatabdi (B), Dart (A), EGA Burke (A), Forrest (A), Lincoln (A), PBW 644 (I), | *Lr1, Lr13* | SR, APR | Bhardwaj et al 2010; Malaker et al 2011; Cuddy et al 2016; Khan et al 2014 |
| Estica (UK, Ho), Sideral (F), Vivant (Ho, F) | *Lr13, Lr14a* | SR | Pathan et al 2006 |
| INIA 66 (USA) | *Lr13, Lr17* | SR | Roelfs et al |
| Contra (G), Riband (UK), Sarka (CR), Kalasz (H) | *Lr13, Lr17b* |  | Pathan et al 2006 |
| Zen (A), Lona (S), Rossini (F) | *Lr13, Lr20* | SR | Cuddy et al 2016 |
| Kanchan (B), Protiva (B) | *Lr13, Lr23* | APR, SR | Malaker et al 2011 |
| Naparoo (A) | *Lr13, Lr24* | SR, APR | Cuddy et al 2016 |
| Amadeus (Au), Apollo (Au, F) | *Lr13, Lr26* |  | Pathan et al 2006 |
| WH 773 (I), | *Lr3, Lr13* | APR, SR | Bhardwaj et al 2010 |
| NIAW 34 (I), VL 832 (I), BH 1146 (USA), Choti Lerma (USA) | *Lr13, Lr34* | APR, SR | Bhardwaj et al 2010; Roelfs et al |
| Sakha 69 (E), Beaufort (A), Derrimut (A), SQP Revenue (A), Ventura (A), Abbot (UK), Rapor (F) | *Lr13, Lr37* | APR,SR | El-Orabey et al 2013; Cuddy et al 2016; Pathan et al 2006 |
| Sids 1 (E) | *Lr13, Lr41* | APR,SR | El-Orabey et al 2013 |
| Zugoly (H) | *Lr13, UGIR* | SR | Pathan et al 2006 |
| Cannon (UK) | *Lr14a, Lr20* | - | Pathan et al 2006 |
| HUW 234 (MALVIYA 234) (I) | *Lr14a, Lr35* | SR, APR | Tomer et al 2014 |
| Renan (F) | *Lr14a, Lr37* | SR | Pathan et al 2006 |
| AC Majestic (C) | *Lr16, Lr13* | SR | Kolmer et al 2002 |
| Toronit (S) | *Lr20, Lr26* | SR | Pathan et al 2006 |
| Orion (A) | *Lr20, Lr37* | SR, APR | Cuddy et al 2016 |
| HD 2824 (I), HUW 549 (I), PBW 519 (I), PBW 500 (I), DWR 162 (I), MACS 2486(I), DL 803-3 (I), HUW 206 (MALVIYA WHEAT206) (I), K 8804 (I), DBW 71 (I) | *Lr23, Lr26* | SR | Bhardwaj et al 2010; Sivasamy 2014 ; Khan et al 2014 |
| HD 2501 (I), HI 977 (I), Kundan (I), UP 262 (I) | *Lr23, Lr34* | APR, SR | Bhardwaj et al 2010; Tomer et al 2014; Sivasamy 2014 |
| Kennedy (A) | *Lr23, Lr46* | SR | Cuddy et al 2016 |
| PBW 703 (I) | *Lr24, Lr28* | - | Gupta et al 2017 |
| Bremer (A), Gazelle (A), Shield (A), Supreme (A) | *Lr24, Lr37* | APR, SR | Cuddy et al 2016 |
| UAS 259 (I), HS 375 (I), UP 2425 (I), UP 2338 (I) | *Lr26, Lr34* | APR, SR | Bhardwaj et al 2010; Sivasamy 2014 |
| Hussar (UK) | *Lr26, Lr37* | - | Pathan et al 2006 |
| Sufi (B), Binzhou 98-6 (Ch), Yannong 15 (Ch), Yan 5286 (Ch), DBW 107 (I), PBW 660 (I) | *Lr3, Lr26* | SR | Malaker et al 2011; Li et al 2018; Khan et al 2014 |
| PBW 343 (I) | *Lr35, Lr26* | SR | Sivasamy 2014 |
| Asta (CR), Pandas (It), Samanta (CR), Tenger (H) | *Lr3a, Lr13* | - | Pathan et al 2006 |
| Centauro | *Lr3a, Lr14a* | - | Pathan et al 2006 |
| EGA Wylie (A) | *Lr3a, Lr17a* | SR | Cuddy et al 2016 |
| Cobra, (A), EGA Gregory (A) | *Lr3a, Lr23* | SR | Cuddy et al 2016 |
| Beckom (A), Gauntlet (A), SF Hekto (A) | *Lr3a, Lr37* | APR, SR | Cuddy et al 2016 |
| Duet (R) | *Lr9, Lr10* | SR | Gul’tyaeva et al 2009 |
| Livingston (A), Sunzell (A) | *Lr1, Lr13, Lr37* | APR, SR | Cuddy et al 2016 |
| Hongtangliangmai (Ch) | *Lr1, Lr20, Lr36* | SR | Zhang et al 2019 |
| VL 822(I), MACS 2496 (I), GW 190 (I), DBW 93 (I), WH 1142 (I) | *Lr1, Lr23, Lr26* | SR | Bhardwaj et al 2010; Sivasamy 2014; Khan et al 2014 |
| Cutlass (A) | *Lr1, Lr24, Lr37* | APR, SR | Cuddy et al 2016 |
| HD 3090 (I) | *Lr1, Lr26, Lr24* | APR, SR | Khan et al 2014 |
| Sourav (B), HPW 42 (I), HS 240 (I) | *Lr1, Lr26, Lr34* | APR, SR | Malaker et al 2011; Sivasamy 2014 |
| Beaufort (UK) | *Lr1, Lr26, Lr37* | - | Pathan et al 2006 |
| Axe (A) | *Lr1, Lr3a or Lr73, Lr13* | APR, SR | Cuddy et al 2016 |
| Vlada (CR) | *Lr1, Lr3a, Lr13* | - | Pathan et al 2006 |
| Boval (S) | *Lr10, Lr13, Lr14a* | - | Pathan et al 2006 |
| Encore (UK), Hunter (UK), Rialto (UK), Siria (CR) | *Lr10, Lr13, Lr26* |  | Pathan et al 2006 |
| HD 2329 (I) | *Lr10, Lr13, Lr34* | APR, SR | Bhardwaj et al 2010 |
| Reaper (UK) | *Lr10, Lr13, Lr37* | - | Pathan et al 2006 |
| AC Splendor (C) | *Lr13, Lr16, Lr34* | APR, SR | Kolmer et al 2002 |
| Arche (F) | *Lr13, Lr17b, Lr37* | - | Pathan et al 2006 |
| HP 1102 (I), HP 1209 (I), HW 741 (I), HW 971 (I) | *Lr13, Lr23, Lr34* | - | Tomer et al 2014 |
| Gemmeiza 3 (E) | *Lr13, Lr24, Lr34* | APR, SR | El-Orabey et al 2013 |
| Gemmeiza 5 (E) | *Lr13, Lr24, Lr35* | APR, SR | El-Orabey et al 2013 |
| Caxton (UK) | *Lr13, Lr26, Lr37* | - | Pathan et al 2006 |
| Mitch (A) | *Lr13, Lr27, Lr31* | SR, APR | Cuddy et al 2016 |
| WH 147 (I) | *Lr13, Lr34, Lr35* | APR, SR | Sivasamy 2014 |
| Lerma Rojo 64A (USA), AC Splendor (USA) | *Lr14a, Lr17, Lr34* | APR, SR | Roelfs et al |
| Yan 861601 (Ch) | *Lr17, Lr26, Lr32* | SR | Li et al 2018 |
| WH 542 (I) | *Lr23, Lr26, Lr34* | APR, SR | Sivasamy 2014 |
| Manning (A) | *Lr23, Lr26, Lr37* | SR, APR | Cuddy et al 2016 |
| HD 2733 (I) | *Lr26, Lr34, Lr35* | APR, SR | Bhardwaj et al 2010 |
| Sunlamb (A) | *Lr27, Lr31, Lr37* | SR, APR | Cuddy et al 2016 |
| Baiheshang (Ch) | *Lr2b, Lr14a, Lr21* | SR | Zhang et al 2019 |
| DBW 110 (I), HD 3086 (I) | *Lr3, Lr10, Lr13* | APR, SR | Khan et al 2014 |
| Parula (I) | *Lr34, Lr46, Lr68* | APR, SR | Tomer et al 2014 |
| Trojan (A) | *Lr3a or Lr23, Lr27, Lr31* | APR, SR | Cuddy et al 2016 |
| Mikon (G), Sparta (CR), MV Palma (H), Fiocco (It), Barra (It) | *Lr3a, Lr13, Lr26* | - | Pathan et al 2006 |
| Equinox (UK) | *Lr3a, Lr26, Lr37* | - | Pathan et al 2006 |
| AC Karma (C) | *LrTb, Lr13, Lr16* | SR | Kolmer et al 2002 |
| Laizhou 953 (Ch) | *Lr1, Lr3, Lr26, Lr32* | SR | Li et al 2018 |
| HS 420 (I) | *Lr1, Lr13, Lr10, Lr34* | APR, SR | Bhardwaj et al 2010 |
| Sunmate (A), Suntime (A), Suntop (A) | *Lr1, Lr27, Lr31, Lr37* | SR, APR | Cuddy et al 2016 |
| Gemmeiza 1 (E) | *Lr12, Lr13, Lr34, Lr36* | APR, SR | El-Orabey et al 2013 |
| HD 2189 (I) | *Lr13, Lr17, Lr23, Lr34* | APR, SR | Tomer et al 2014 |
| Sakha 92 (E) | *Lr13, Lr34, Lr35, Lr36* | APR, SR | El-Orabey et al 2013 |
| Sakha 8 (E) | *Lr9, Lr12, Lr13, Lr24, Lr34* | APR, SR | El-Orabey et al 2013 |
| Gemmeiza 9 (E) | *Lr9, Lr13, Lr24, Lr34, Lr41* | APR, SR | El-Orabey et al 2013 |
| HS 420 (I) | *Lr1, Lr3, Lr10, Lr13, Lr23, Lr26* | SR | Tomer et al 2014 |
| BARI Gom-26 (B) | *Lr10, Lr13, Ltn* | APR, SR | Malaker et al 2011 |
| Scepter (A) | *Lr13, Lr20, Lr37, Lr46* | SR, APR | Cuddy et al 2016 |
| FWW2(INGR17010) (I) | *Lr19, Lr24, Lr26, Sr24, Sr25, Sr31, Yr9* | APR, SR | Bhardwaj et al 2019 |
| FLW24 (07005) (I) | *Lr19, Lr26, Sr25, Sr31, Yr9, Yr27* | APR, SR | Bhardwaj et al 2019 |
| FLW4 (03016) (I) | *Lr24, Lr26, Sr2, Sr24, Sr31, Yr9* | APR, SR | Bhardwaj et al 2019 |
| FLW2 (03014) (I) | *Lr24, Lr26, Sr24, Sr31, Yr9, Yr27* | APR, SR | Bhardwaj et al 2019 |
| HW 1085 (I) | *Lr24, Sr24, Sr31* | APR | Sivasamy 2014 |
| FLW33(INGR17042) (I) | *Lr24, Sr32* | APR | Bhardwaj et al 2019 |
| FLW31(INGR17040) (I) | *Lr24, Sr43* | APR | Bhardwaj et al 2019 |
| FLW20 (07001) (I) | *Lr26, Lr19, Lr24, Sr31, Yr9, YrPBW343* | APR, SR | Bhardwaj et al 2019 |
| FLW21(INGR17008) (I) | *Lr26, Lr24, Sr24, Sr31, Yr9, Yr15* | APR, SR | Bhardwaj et al 2019 |
| FLW30 (08003) (I) | *Lr26, Lr28, Sr31, Yr9, Yr15* | SR | Bhardwaj et al 2019 |
| FLW29 (08002) (I) | *Lr26, Lr28, Sr31, Yr9, YrCD* | SR | Bhardwaj et al 2019 |
| FLW22(INGR17009) (I) | *Lr26, Lr28, Sr31, Yr9, YrChina-84* | SR | Bhardwaj et al 2019 |
| FLW15 (05006) (I) | *Lr26, Lr32, Sr31, Yr9, YrPBW343* | APR, SR | Bhardwaj et al 2019 |
| FLW18(INGR17070) (I) | *Lr26, Lr39, Sr31, Yr9, Yr (PBW343)* | SR | Bhardwaj et al 2019 |
| FLW10(INGR17006) (I) | *Lr26, Sr31, Yr9, Yr10* | SR | Bhardwaj et al 2019 |
| FLW16(INGR17007) (I) | *Lr26, Sr31, Yr9, Yr5* | SR | Bhardwaj et al 2019 |
| FKW1 (06004) (I) | *Lr26, Sr31, Yr9, YrChina-84* | SR | Bhardwaj et al 2019 |
| FLW25(07006) (I) | *Lr28, Lr26, Sr31, Yr9, Yr27* | SR | Bhardwaj et al 2019 |
| FLW26 (07007) (I) | *Lr42, Lr26, Sr31, Yr9, Yr27* | SR | Bhardwaj et al 2019 |
| FLW27 (07008) (I) | *Lr45, Lr26, Sr31, Yr9, Yr27* | SR | Bhardwaj et al 2019 |
| Pavon 76 (I) | *Lr46, Yr29* | APR | Tomer et al 2014 |
| PAU 16055 (I), PAU 16062 (I) | *Lr57, Yr40* |  | Gupta et al 2017 |
| PBW 723 (Unnat PBW 343) (I) | *Lr57/Yr40, Lr37/Yr17* | APR, SR | IIWBR Karnal |
| RL 6077 (I) | *Lr67, Yr46* | APR | Tomer et al 2014 |
| FLW6 (04011) (I) | *Lr9, Lr24, Sr2, Sr24* | APR, SR | Bhardwaj et al 2019 |

^*^SR: Seedling resistance, ^**^APR: Adult plant resistance; ^***^

ASR: All stage resistance. The abbreviations mentioned in the parenthesis against each variety indicate the countries of origin of the respective varieties.

**ESM Table 5:** A summary of reports of introgression/pyramiding of Lr genes in wheat using MAS.

| Lr genes | Donor genotype | Recipient genotype | Linked marker  (Name and type) | Reference |
| --- | --- | --- | --- | --- |
| *Lr1, Lr9, Lr24* and *Lr47* | Thatcher NILs | Bolero, ColWorito, Serio and Spada | *Lr24* (*CH51* and *SCH52*; SCAR ); *Lr1* (*pTAG621*; STS); *Lr9* (*J13*; STS), *Lr47* ( *PS10*; CAPS) | Nocentoet al., 2007 |
| *Lr9* and *Lr24* | Thatcher NILs | Swiss winter wheat, "Arina" | *Lr9* (*J13* and *SCS5*; STS) and *Lr24* (*J09*and *H05*; STS) | Moulletet al., 2009 |
| *Lr19* and *Lr24* | - | HD2733 | *Lr19* (*xwmc221*; SSR) and *Lr24* (*SCS1302*; SCAR) and SCAR | Singh et al., 2017 |
| *Lr41* and *Pm21* | KS90WGRC10 (*Lr41*) and Yangmai 5 (*Pm21*) | Polish wheat cv Nadobna | *Lr41* (*Gdm35*, *Barc124*, *Gwm261*, *Gwm296*, *Gwm210;* SSR) and *Pm21*(*SCAR1250*, *SCAR1400;* SCAR) | Pietrusinskaet al., 2011 |
| *L37, Lr24* and *Lr76* | Improved lines of PBW343 | PBW343 | *Lr37* (*VENTRIUP-LN2*), *Lr24* (*xbarc70*; SSR), *Lr76* (*Lr57-Yr40_CAPS*; CAPS) | Gautam et al., 2020 |
| *Lr24* and *Lr28* | NILs of PBW343 for both the genes | PBW343 | *Lr24* (*S73_719_*_;_ SCAR) and *Lr28 S421_570_*; SCAR) | Chhunejaet al., 2011 |
| *Lr9, Lr24* and *Lr28* | NILs of HD2329 for each of the three genes | HD2329 | *Lr9* (*SCS1302_607_* ;SCAR), *Lr24* (*SCS421_570_*; SCAR), *Lr28* (*SCSS5_550_*;SCAR) | Charpeet al., 2012 |
| *Lr24* and *Lr28* | Sunstar (Lr24 and Yr15), HW2033 (Lr28) | HD2877 | *Lr24* (*SCS1320_607_* ; SCAR) and *Lr28* (*xgwm273*; SSR)and SSR | Revathiet al., 2010 |
| *Lr24* and *Lr48* | CSP44 (Lr48) and PBW343 (Lr24) | PBW343 | *Lr24* (*SCS1320_607_*; SCAR) and *Lr48* (*S3_450_*and *S336_775_* ; RAPD) | Samsampouret al., 2009 |
| *Lr19* | Agrus and Sunnan | Sofia, Simona and Livia | Endopeptidase allele *Ep-D1c;* gene specific | Slikovaet al., 2003 |
| *Lr19 for leaf rust along-with Sr26* and *Yr10* | H2687 and exotic variety Avotec | HD2932 | *Lr19* (*xwmc221*; SSR) *Yr10* (*xpsp3000*; SSR) and *Sr26* (*Sr26#43, BE518379*; SCAR) | Mallicket al., 2015 |
